# Plasma BDNF Is Associated with Age-Related White Matter Atrophy but Not with Cognitive Function in Older, Non-Demented Adults

**DOI:** 10.1371/journal.pone.0035217

**Published:** 2012-04-16

**Authors:** Ira Driscoll, Bronwen Martin, Yang An, Stuart Maudsley, Luigi Ferrucci, Mark P. Mattson, Susan M. Resnick

**Affiliations:** 1 Laboratory of Behavioral Neuroscience, National Institute on Aging, Baltimore, Maryland, United States of America; 2 Psychology Department, University of Wisconsin-Milwaukee, Milwaukee, Wisconsin, United States of America; 3 Laboratory of Clinical Investigation, National Institute on Aging, Baltimore, Maryland, United States of America; 4 Laboratory of Neurosciences, National Institute on Aging, Baltimore, Maryland, United States of America; 5 Clinical Research Branch, National Institute on Aging, Baltimore, Maryland, United States of America; Chiba University Center for Forensic Mental Health, Japan

## Abstract

Brain derived neurotrophic factor (BDNF) seems to be involved in regulation of synaptic plasticity and neurogenesis. BDNF plasma and serum levels have been associated with depression, Alzheimer's disease, and other psychiatric and neurodegenerative disorders. In a community sample, drawn from the Baltimore Longitudinal Study of Aging (BLSA), we examined whether BDNF plasma concentration was associated with rates of age-related change in cognitive performance (n = 429) and regional brain volume (n = 59). Plasma BDNF levels, which were significantly higher in females (p<0.05), were not associated with either concurrent cognitive performance or rates of age-related change in performance across cognitive domains (*p*'s>0.05). Sex differences in the relationship between BDNF and the trajectories of regional brain volume changes were observed for the whole brain and frontal white matter volumes (*p*<0.05), whereby lower plasma BDNF was associated with steeper volume decline in females but not males. Together, our findings contribute to furthering the understanding of the relationships between plasma BDNF, structural brain integrity and cognition. Potential mechanisms mediating these relationships merit further investigation.

## Introduction

Alzheimer's disease (AD) is a fatal neurodegenerative disorder characterized by the progressive loss of both grey and white matter, and cognitive impairment. Understanding pathogenic mechanisms and identifying biological risks is imperative to the discovery of biomarkers for differential diagnosis and treatment, as well as pharmacological targets. Much effort has focused on the role of neurotrophic factors, particularly brain-derived neurotrophic factor (BDNF), in both the pathogenesis and the disease course of AD [1], [2].

BDNF is expressed throughout the brain, especially in the prefrontal cortex and the hippocampus [3]. Reduced BDNF levels in various brain regions have been implicated in the pathogenesis of neurodegenerative and psychiatric disorders [2]. Although human findings derive primarily from investigations of an allelic variant of BDNF (*Val66Met*), cumulative physiological, genetic and neuroimaging findings all favor the idea that BDNF influences cognitive function [4]. Moreover, *Val66Met* has been related to smaller hippocampal volumes [3], [5], [6] , which in turn are associated with worse memory [7] and more rapid conversion to dementia [8]. The mechanisms underlying these BDNF associations with brain function and structure, however, remain unclear. It is also unclear whether there is a relationship between BDNF genotypes and BDNF measured in plasma.

There are only a limited number of studies examining the relationships between polymorphisms in BDNF gene and plasma BDNF. In rhesus monkeys, a Val to Met transition in the pro-BDNF domain, similar to a well-described variant in the human gene, seems to affect peripheral levels of BDNF [9]. However, in a relatively large community sample of healthy adults (N = 391) drawn from the Baltimore Longitudinal Study of Aging BDNF plasma levels were not associated with the Val66Met variant in BDNF gene in either men or women [10]. The rs7124442T/rs11030102C/rs11030119G haplotype in the BDNF gene was associated with higher BDNF plasma levels, albeit in eating disorder patients while a similar but non-significant trend was observed in control group consisting of sibling pairs discordant for the disorder [11].

It has become apparent that BDNF is present outside the central nervous system (CNS) and circulates systemically. Despite evidence from both human and non-human studies demonstrating the role for BDNF in the regulation of energy metabolism and the cardiovascular system [12], [13], little is known about the role of plasma BDNF in pathological states. The identification of potential biomarkers that can be measured non-invasively *in-vivo* is of great potential for diagnosis and treatment. However, greater understanding of the role of these biomarkers in normal aging, and as distinct from explicit disease or pathology, is a pre-requisite to the development of rational approaches to prevention and treatment. Limited existing literature suggests that low BDNF levels in the cerebrospinal fluid (CSF) are associated with cognitive decline, both cross-sectionally and longitudinal [14]; however, little is known about the association of peripheral BDNF levels with either brain structure or cognition in late life.

To elucidate the role for BDNF in structural and functional brain integrity during aging, we measured plasma BDNF in a non-clinical sample of older, community dwelling individuals who were prospectively followed through the Baltimore Longitudinal Study of Aging (BLSA). We hypothesized that lower levels of plasma BDNF would be associated with both steeper regional brain volume decline and decline in performance on cognitive tasks dependent on those regions.

## Results

Participant characteristics are summarized in Table 1. The age range was from 50 to 97.5 years (56–90 for the neuroimaging (NI) sub-sample). There were no significant differences in age, education, race, follow-up interval or the number of assessments between the whole sample and the NI subsample. Results are reported as significant if p≤0.05, although with Bonferroni correction for multiple comparisons, results would be considered significant at p≤0.0013.

**Table 1:** Sample Characteristics.

	N	Baseline Age	Follow-up Interval	Number of Assessments	Plasma BDNF	Education	Race B/W
**Sample**	429	61.9 (9.1)	9.3 (4.5)	5.3 (3.2)	770 (570)	16.3 (2.6)	121/308
**Males**	216	63.1 (9.3)	9.5 (4.6)	5.4 (3.2)	647 (526)	16.5 (2.7)	47/169
**Females**	213	60.6 (8.9)	9.1 (8.9)	5.2 (3.2)	896 (586)	16.1 (2.5)	74/139

Mean (SD).

### Plasma BDNF levels are greater in females compared to males

BDNF plasma concentration in our sample averaged 759 pg/ml (SD = 551; range 38–2475). Older age was significantly associated with lower plasma BDNF (F_(1,325)_ = 5.39, p = 0.02). Plasma BDNF levels were significantly higher in females (p<0.05) than in males. There were no significant relationships between plasma BDNF and race (p>0.8) or education (p>0.9).

### Plasma BDNF does not predict cognitive performance

No significant relationships were observed between plasma BDNF and neuropsychological test scores obtained at the same visit as the blood sample (all p>0.1). Similarly, there were no significant relationships between plasma BDNF and mean cognitive scores on each test across all available visits (all p>0.09). The lack of significant associations held when examining males and females separately (p≥0.05). Plasma BDNF levels did not predict the rates of change with age in performance on any of the cognitive domains examined (p's>0.1).

### Higher plasma BDNF levels are associated with slower change in white matter volume with age (Table 2)

**Table 2:** Cross-sectional (main) effects of BDNF on regional brain volume and longitudinal brain volume changes (annual rates in cm^3^) in relation to plasma BDNF.

	Cross-sectional Effects		Longitudinal Effects	
REGION	BDNF	Sex* BDNF	BDNF* interval	Sex*BDNF *interval
Whole Brain	0.77 (1.28)	0.46 (2.61)	0.06 (0.05)	**−0.22** * **(0.11)**
vCSF	0.03 (0.34)	0.66 (0.70) 0.35	0.014 (0.01)	0.04 (0.03)
GM	−0.54 (0.74)	−2.68 (1.52)	−0.044 (0.05)	−0.12 (0.09)
WM	1.21 (0.89)	3.03 (1.82)	0.07 (0.04)	−0.033 (0.09)
**Gray Matter:**				
Frontal	−0.10 (0.25)	−0.79 (0.52)	−0.02 (0.02)	−0.04 (0.03)
Temporal	−0.17 (0.18)	−0.56 (0.37)	−0.02 (0.02)	−0.01 (0.03)
Parietal	−0.26 (0.16)	−0.57 (0.34)	−0.01 (0.01)	0.0004 (0.02)
Occipital	−0.08 (0.13)	−0.16 (0.27)	0.003 (0.01)	−0.013 (0.02)
**White Matter:**				
Frontal	0.58 (0.39)	0.80 (0.79)	0.001 (0.02)	**−0.09** ** **(0.03)**
Temporal	0.20 (0.23)	0.49 (0.48)	**0.04** ** **(0.01)**	−0.01 (0.03)
Parietal	0.18 (0.19)	0.69 (0.39)	0.02 (0.01)	0.02 (0.02)
Occipital	**0.26** * **(0.12)**	**0.71** ** **(0.25)**	0.02 (0.01)	−0.01 (0.02)
**Other Regions:**				
Orbito-frontal	−0.03 (0.06)	−0.07 (0.11)	−0.002 (0.004)	−0.01 (0.01)
Hippocampus	−0.004 (0.01)	−0.012 (0.03)	0.001 (0.001)	0.002 (0.002)
Cingulate Gyrus	−0.02 (0.06)	−0.02 (0.13)	−0.003 (0.01)	0.01 (0.01)
Precuneus	−0.003 (0.02)	−0.05 (0.04)	0.001 (0.001)	−0.003 (0.002)

Results are expressed as β-coefficients (S.E.).

** p<0.01.

* p<0.05.

A significant BDNF by sex interaction was observed for the trajectories of frontal WM volume (F(1,360) = 7.52, p = 0.006; Table 1), where a strong but marginally significant trend was present for slower rates of frontal WM decline in association with higher BDNF levels in females (p = 0.05). A marginally significant trend was noted for slower rates of whole brain volume changes with age in association with higher BDNF levels in females (p = 0.05). Cross-sectional analyses revealed a general lack of relationship between any of the regional brain volumes examined and plasma BDNF (*p*≥0.05), except for occipital WM (F(1,59.7) = 7.47, p = 0.008) where higher plasma BDNF was associated with larger volumes in males (p = 0.002).

## Discussion

Our results are in accord with the existing literature reporting lower serum and plasma BDNF levels with increasing age [12], [15], [16], with significantly higher levels observed in women compared to men [12], [16]–[18]. We found that lower plasma BDNF was associated with steeper rates of age-related regional brain volume atrophy. Contrary to our expectations, however, we observed no associations between circulating plasma BDNF levels and either concurrent cognitive performance or the rate of change in cognitive performance with age.

Human genetic studies implicate BDNF gene involvement in cognitive function, specifically memory and executive function [4], [19]. Evidence also suggests that higher serum BDNF levels are associated with better cognitive performance in healthy older adults [15]. We found no associations between plasma BDNF levels and either concurrent cognitive performance, mean cognitive performance across all available assessments, or the rate of change in cognition with age across domains in a large sample of healthy, older individuals. The lack of associations between plasma BDNF and cognitive performance in our sample stands in contrast to the only existent study exploring this relationship in a large Eastern Finnish sample (N = 1389). In the Finnish sample, the risk for worse performance on tests tapping memory and general cognitive function increased with a drop in plasma BDNF, but the associations were only present for women and not men [17]. Li and colleagues [14] however, reported that lower CSF BDNF concentration was associated with poorer immediate and delayed recall measured at baseline and three years later. Given the general lack of studies on this topic, discrepancies between our findings and those of the two above-mentioned studies may reflect methodological differences in general and more specifically, differences in participant sampling. Cross-sectional studies of normal individuals may be contaminated with individuals in prodromal stages of impairment that goes unnoticed due to inherent lack of prospective information. It is possible that plasma BDNF may have little effect on cognitive performance within the normal range of function in non-demented, healthy older adults, whereby considerable decrease in BDNF may only be observable in the late stage of cognitive decline or dementia, as is the case with bipolar disorder for example [20].

We found that lower plasma BDNF is associated with steeper regional brain volume decline. In our sample, lower BDNF levels predicted steeper white matter atrophy and this relationship was present in women but not men. The mechanisms underlying the apparent sex differences in findings remain elusive, however, ovarian steroids have been implicated as a modulating factor on BDNF levels [21]. Effects of estrogen on brain BDNF have been well documented in rodents [22], [23], albeit mice used in these studies were young.

Our findings differ from the existing literature, which reports significant associations of gray matter and the hippocampus with either serum BDNF [7] or Val66Met polymorphism [24], [25]. Basic research, however, suggests a role for BDNF in white matter neuroprotection, whereby BDNF application promotes secondary decrease of white matter lesion size even though it does not prevent lesions from appearing [26]. Moreover, BDNF has been implicated as an important factor associated with white matter volume in patients with multiple sclerosis independent of BDNF Val66Met (rs6265) genotype [27].

Our study is not without limitations. There is currently no reliable method to detect both pro-BDNF and BDNF in human plasma or serum, as there is antibody cross-reactivity between pro-BDNF and BDNF due to their high sequence homology. Thus, at this point in time, it is difficult to determine whether changes in plasma or serum BDNF reflect pro-BDNF, BDNF or both. Novel assays need to be developed that can accurately and quantitatively distinguish between pro-BDNF and BDNF. Moreover, the present sample is not population-based; participants are highly educated and predominantly Caucasian. The relative homogeneity of the sample, however, may be seen as an advantage because the majority of our sample has good access to medical care and has remained relatively healthy over the follow-up interval. Moreover, average BDNF plasma values vary widely across studies, most likely owing to the fact that the concentration of BDNF in plasma is state-dependent [28]–[30]. Methodological differences notwithstanding, the average BDNF plasma concentration in our study is similar to that of other non-clinical samples [16], [17]. These limitations, however, should not undermine the unique aspects of our study, namely a large number of elderly individuals who were prospectively followed for many years on an annual basis, extensively characterized, and studied with state-of-the art image processing methods. Moreover, we characterize age-related changes in many of the regional brain volumes that have not been previously investigated in relation to plasma BDNF.

Another issue in interpreting our results is the analysis of a number of cognitive domains and a number of brain volume measurements without adjustment for multiple comparisons. Given the relative dearth of studies investigating associations between plasma BDNF and cognitive and brain outcomes, our exploratory investigation in a well-characterized longitudinal sample should provide directions for future research. In addition, our long follow-up interval ensures great power for detecting even small changes with age, which would not be possible in a cross-sectional design.

To our knowledge, this study is the first longitudinal study exploring associations between plasma BDNF levels, brain volume and cognitive function in healthy older individuals. Overall, our results suggest a role for BDNF in structural brain integrity, but no direct associations with cognitive function or change in cognition with age. Together, our findings from the BLSA cohort contribute to furthering the understanding of the relationships between plasma BDNF concentration, brain volume integrity and cognitive function with age. Further research is needed in order to understand not only the role for BDNF in brain structure and function, but also the relationship between peripheral BDNF (both plasma and serum) and its genetic variants. Additional research is needed to demonstrate the mechanisms underlying the relationship between plasma BDNF and brain volume integrity, as well as the sex differences that might be moderating the relationship, especially considering that current knowledge is primarily based on animal studies and human genetic studies.

## Materials and Methods

### Participants

The sample was drawn from the BLSA [31], an ongoing multidisciplinary study of community-dwelling volunteers. At each evaluation, participants underwent neuropsychological testing, a neurological exam, interval medical history, medication review, and a structured informant and subject interview detailed elsewhere [31], [32]. Inclusion criteria are good general health. Specific exclusionary criteria at enrollment for the BLSA NI sub-study included a history of CNS disease (dementia, stroke, Parkinson's disease, epilepsy, and other neurological conditions), severe cardiac disease (including myocardial infarction, coronary bypass surgery, or angioplasty), and metastatic cancer. All individuals who went on to develop cognitive impairment or dementia, based on standardized consensus diagnostic procedures for the BLSA [33] using Peterson criteria for MCI [34], were excluded from analyses. BLSA studies were approved by the local institutional review boards of both the National Institute on Aging and Johns Hopkins Medical Institutions, and all participants gave written informed consent prior to each assessment.

Blood samples were obtained from 429 participants (213 F/216 M; 64% Caucasian) during regularly scheduled visits from January 2007 to June 2008. Participants ranged in age from 50 to 98 years (M = 62, SD = 9.1 and were on average college-educated (M = 16.3 years of education, SD = 2.6). A subset of 59 participants, who were characteristically similar to the larger BLSA sample, also underwent prospective, annual neuroimaging (NI) assessments for up to 10 years. Sample characteristics are summarized in Table 1.

### Measurement of plasma BDNF concentration

Blood samples were obtained in the morning after an overnight fast. Blood samples were centrifuged at 3000 rpm for 30 minutes at 4°C. Plasma was carefully collected and was snap frozen on dry ice and subsequently stored at −80°C, until used for further analyses. BDNF plasma levels were measured using a commercially available ELISA kit (Promega, Madison, WI; sensitivity range 7.8–500 pg/ml; inter-assay variation = 8.8% (low concentration), 2.9% (medium concentration), and 2.2% (high concentration) as described in more detail previously [35]–[37]. Briefly, the plasma samples were diluted 1∶5 in block and sample buffer provided by the kit. The BDNF plate was coated with primary BDNF antibody overnight, the following day block and sample buffer were added to each well for 1 hour, and subsequently the standards and samples were added for 2 hours. Thereafter, anti-human BDNF pAb secondary antibody was added for 2 hours and the anti-Ig Y HRP conjugate was added for 1 hour. Then, TMB One solution was added to each well for 5 minutes and the reaction was stopped with hydrochloric acid. The plate was analyzed within 30 minutes at 450 nm.

### Neuropsychological assessment

Assessments spanned five different cognitive domains: memory, word knowledge, attention, executive function, and visuo-spatial abilities across a battery of 12 neuropsychological tests. Memory was assessed using the California Verbal Learning Test (CVLT) [38], Benton Visual Retention Test (BVRT) [39] and Cued Selective Reminding Test (CSR) [40]. Primary Mental Abilities Vocabulary (PMA) [41] assessed word knowledge. Verbal fluency was assessed by letter fluency (FAS) and semantic fluency [42]. The Digit Span subtest from the Wechsler Adult Intelligence Scale-Revised [43] and the Trail Making Test [44], parts A and B, assessed attention and executive function. Digits Forward and Trails A assessed attention. Digits Backward, Trails B, and Verbal Fluency (categories and letters) measured executive function. The Card Rotations Test [45] assessed visuo-spatial function.

### MRI acquisition

Scanning was performed on a GE Signa 1.5 Tesla scanner (Milwaukee, WI) using a high-resolution volumetric spoiled-grass (SPGR) axial series (TR = 35 ms, TE = 5 ms, FOV = 24 cm, flip angle = 45°, matrix = 256×256, NEX = 1, voxel dimensions 0.94×0.94×1.5 mm).

### MRI image analysis

Image processing procedures have been previously described and validated [46]–[48]. Briefly, images were corrected for head tilt and rotation, and reformatted parallel to the anterior-posterior commissure plane. Extracranial tissue was removed using a semi-automated procedure followed by manual editing. Next, images were segmented into white matter (WM), gray matter (GM), and cerebrospinal fluid (CSF). The final step involved stereotaxic normalization and tissue quantitation for specific regions of interest. A template-based deformation approach was employed, using the ICBM standard MRI (Montreal Neurologic Institute) as the template and a hierarchical elastic matching algorithm for deformation and regions of interest determination [49]. All images were normalized individually to the same template. Voxel-based analysis utilized the RAVENS approach [47]. Intracranial volume (ICV) is determined using the template warping algorithm modified for head image registration. First, the ICV in the template is manually and carefully delineated by an expert. Then, the template with its ICV mask is warped to the space of each individual head. Finally, the warped ICV mask of the template is used to directly extract the ICV of the individual.

### Statistical analysis

Linear mixed effects models (i.e., random effects models) [50], [51] were performed using SAS 9.1 (SAS Institute Inc.; Cary, NC) to investigate the associations between BDNF and cross-sectional and longitudinal measures of cognitive function and regional brain volume changes. Pair-wise multiple comparisons with Tukey adjustments were carried out to compare sample characteristics.

Regional brain volumes and performance on individual neuropsychological tests served as dependent variables. For analysis of BDNF in relation to cognitive change, the fixed effects included the following terms: intercept, sex, age at the last measurement (agelast), BDNF, interval (from last MRI), sex*BDNF, sex*interval, agelast*interval, BDNF*interval and sex*BDNF*interval. Regional brain volume analyses followed the same model, but included intra-cranial volume as an additional covariate.

We used effect coding for sex (−0.5 for female and 0.5 for male). BDNF was centered at the sample mean and divided by 100. Age at the last measurement was centered at 75. This model formulation allowed us to investigate cross-sectional (concurrent) BDNF effects on brain volumes and cognitive measures and BDNF effects on the longitudinal rates of change in brain volume and cognition simultaneously after adjusting for age and sex.

Model reductions were conducted on random effects, which included intercept and interval. Non-significant random effects were dropped from the model. No model reductions were performed on the fixed effects [52].

## References

[1] Mattson MP, Maudsley S, Martin B (2004a). A neural signaling triumvirate that influences ageing and age-related disease: Insulin/IGF-1, BDNF and serotonin.. Ageing Res Rev.

[2] Mattson MP, Maudsley S, Martin B (2004b). BDNF and 5-HT: a dynamic duo in age-related neuronal plasticity and neurodegenerative disorders.. Trends Neurosci.

[3] Pezawas L, Verchinski BA, Mattay VS, Callicott JH, Kolachana BS (2004). The brain-derived neurotrophic factor val66met polymorphism and variation in human cortical morphology.. J Neurosci.

[4] Savitz J, Solms M, Ramesar R (2006). The molecular genetics of cognition: dopamine, COMT and BDNF.. Genes Brain Behav.

[5] Bueller JA, Aftab M, Sen S, Gomez-Hassan D, Burmeister M (2006). BDNF Val66Met allele is associated with reduced hippocampal volume in healthy subjects.. Biol Psychiatry.

[6] Szeszko PR, Lipsky R, Mentschel C, Robinson D, Gunduz-Bruce H (2005). Brain-derived neurotrophic factor val66met polymorphism and volume of the hippocampal formation.. Mol Psychiatry.

[7] Erickson KI, Prakash RS, Voss MW, Chaddock L, Heo S (2010). Brain-derived neurotrophic factor is associated with age-related decline in hippocampal volume.. J Neurosci.

[8] Grundman M, Sencakova D, Jack CR, Petersen RC, Kim HT (2002). Brain MRI hippocampal volume and prediction of clinical status in a mild cognitive impairment trial.. J Mol Neurosci.

[9] Cirulli F, Reif A, Herterich S, Lesch KP, Berry A (2011). A novel BDNF polymorphism affects plasma protein levels in interaction with early adversity in rhesus macaques.. Psychoneuroendocrinology.

[10] Terracciano A, Martin B, Ansari D, Tanaka T, Ferrucci L (2010). Plasma BDNF concentration, Val66Met genetic variant and depression-related personality traits.. Genes Brain Behav.

[11] Mercader JM, Ribasés M, Gratacòs M, González JR, Bayés M (2007). Altered brain-derived neurotrophic factor blood levels and gene variability are associated with anorexia and bulimia.. Genes Brain Behav.

[12] Golden E, Emiliano A, Maudsley S, Windham BG, Carlson OD (2010). Circulating brain-derived neurotrophic factor and indices of metabolic and cardiovascular health: data from the Baltimore Longitudinal Study of Aging.. PLoS One.

[13] Johnson JB, Summer W, Cutler RG, Martin B, Hyun DH (2007). Alternate day calorie restriction improves clinical findings and reduces markers of oxidative stress and inflammation in overweight adults with moderate asthma.. Free Radic Biol Med.

[14] Li G, Peskind ER, Millard SP, Chi P, Sokal I (2009). Cerebrospinal fluid concentration of brain-derived neurotrophic factor and cognitive function in non-demented subjects.. PLoS One.

[15] Gunstad J, Benitez A, Smith J, Glickman E, Spitznagel MB (2008). Serum brain-derived neurotrophic factor is associated with cognitive function in healthy older adults.. J Geriatr Psychiatry Neurol.

[16] Lommatzsch M, Zingler D, Schuhbaeck K, Schloetcke K, Zingler C (2005). The impact of age, weight and gender on BDNF levels in human platelets and plasma.. Neurobiol Aging.

[17] Komulainen P, Pedersen M, Hänninen T, Bruunsgaard H, Lakka TA (2008). BDNF is a novel marker of cognitive function in ageing women: the DR's EXTRA Study.. Neurobiol Learn Mem.

[18] Trajkovska V, Marcussen AB, Vinberg M, Hartvig P, Aznar S (2007). Measurements of brain-derived neurotrophic factor: methodological aspects and demographical data.. Brain Res Bull.

[19] Rybakowski JK, Borkowska A, Skibinska M, Hauser J (2006). Illness-specific association of val66met BDNF polymorphism with performance on Wisconsin Card Sorting Test in bipolar mood disorder.. Mol Psychiatry.

[20] Kapczinski F, Dal-Pizzol F, Teixeira AL, Magalhaes PV, Kauer-Sant'Anna M (2011). Peripheral biomarkers and illness activity in bipolar disorder.. J Psychiatr Res.

[21] Young EA (1998). Sex differences in the HPA axis: implications for psychiatric disease.. J Gend Specif Med.

[22] Meltser I, Tahera Y, Simpson E, Hultcrantz M, Charitidi K (2008). Estrogen receptor beta protects against acoustic trauma in mice.. J Clin Invest.

[23] Sasahara K, Shikimi H, Haraguchi S, Sakamoto H, Honda S (2007). Mode of action and functional significance of estrogen-inducing dendritic growth, spinogenesis, and synaptogenesis in the developing Purkinje cell.. J Neurosci.

[24] Savitz JB, Drevets WC (2009). Imaging phenotypes of major depressive disorder: genetic correlates.. Neuroscience.

[25] van Haren NE, Bakker SC, Kahn RS (2008). Genes and structural brain imaging in schizophrenia.. Curr Opin Psychiatry.

[26] Husson I, Rangon CM, Lelièvre V, Bemelmans AP, Sachs P (2005). BDNF-induced white matter neuroprotection and stage-dependent neuronal survival following a neonatal excitotoxic challenge.. Cereb Cortex.

[27] Weinstock-Guttman B, Zivadinov R, Tamaño-Blanco M, Abdelrahman N, Badgett D (2007). Immune cell BDNF secretion is associated with white matter volume in multiple sclerosis.. J Neuroimmunol.

[28] Brunoni AR, Lopes M, Fregni F (2008). A systematic review and meta-analysis of clinical studies on major depression and BDNF levels: implications for the role of neuroplasticity in depression.. Int J Neuropsychopharmacol.

[29] Lee HY, Kim YK (2008). Plasma brain-derived neurotrophic factor as a peripheral marker for the action mechanism of antidepressants.. Neuropsychobiology.

[30] Piccinni A, Marazziti D, Del Debbio A, Bianchi C, Roncaglia I (2008). Diurnal variation of plasma brain-derived neurotrophic factor (BDNF) in humans: an analysis of sex differences.. Chronobiol Int.

[31] Shock NW, Greulich RC, Andres R, Arenberg D, Costa PT (1984). Normal human aging: The Baltimore longitudinal study of aging..

[32] Zonderman AB, Giambra LM, Arenberg D, Resnick SM, Costa PT (1995). Changes in immediate visual memory predict cognitive impairment.. Archiv Clin Neuropsychol.

[33] Driscoll I, Resnick SM, Troncoso JC, An Y, O'Brien R (2006). Impact of Alzheimer's pathology on cognitive trajectories in nondemented elderly.. Ann Neurol.

[34] Petersen RC (2004). Mild cognitive impairment as a diagnostic entity.. J Intern Med.

[35] Martin B, Pearson M, Kebejian L, Golden E, Keselman A (2007). Sex-dependent metabolic, neuroendocrine, and cognitive responses to dietary energy restriction and excess.. Endocrinology.

[36] Carlson O, Martin B, Stote KS, Golden E, Maudsley S (2007). Impact of reduced meal frequency without caloric restriction on glucose regulation in healthy, normal-weight middle-aged men and women.. Metabolism.

[37] Harvie MN, Pegington M, Mattson MP, Frystyk J, Dillon B (2011). The effects of intermittent or continuous energy restriction on weight loss and metabolic disease risk markers: a randomized trial in young overweight women.. Int J Obes (Lond).

[38] Delis DC, Kramer JH, Kaplan E, Ober BA (1987). California Verbal Learning Test - research edition.

[39] Benton AL (1968). Differential behavioral effects in frontal lobe disease.. Neuropsychologia.

[40] Grober E, Buschke H (1987). Genuine memory deficits in dementia.. Develop Neuropsychol.

[41] DeFries JC, Johnson RC, Kuse AR, McClearn GE, Polovina J (1974). Near identity of cognitive structure in two ethnic groups.. Science.

[42] Newcombe F (1969). Missile wounds of the brain. A study of psychological deficits.

[43] Wechsler D (1981). Wechsler Adult Intelligence Scale – Revised.

[44] Reitan R (1992). Trail Making Test. Manual for administration and scoring.

[45] Wilson JR, Vandenberg SG, McGill TE, Dewsbury DA, Sachs BD (1978). Sex differences in cognition: evidence from the Hawaii Family Study.. Sex and Behavior.

[46] Davatzikos C, Genc A, Xu D, Resnick SM (2001). Voxel-based morphometry using the RAVENS maps: methods and validation using simulated longitudinal atrophy.. Neuroimage.

[47] Goldszal AF, Davatzikos C, Pham DL, Yan MX, Bryan RN (1998). An image-processing system for qualitative and quantitative volumetric analysis of brain images.. J Comput Assist Tomogr.

[48] Resnick SM, Goldszal AF, Davatzikos C, Golski S, Kraut MA (2000). One-year age changes in MRI brain volumes in older adults.. Cereb Cortex.

[49] Shen D, Davatzikos C (2002). HAMMER: hierarchical attribute matching mechanism for elastic registration.. IEEE Trans Med Imaging.

[50] Hartley HO, Rao JNK (1967). Maximum likelihood estimation for the mixed analysis of variance model.. Biometrika.

[51] Laird NM, Ware JH (1982). Random-effects models for longitudinal data.. Biometrics.

[52] Harrell F (2001). Regression Modeling Strategies.

